# Using Hybrid Angle/Distance Information for Distributed Topology Control in Vehicular Sensor Networks

**DOI:** 10.3390/s141120188

**Published:** 2014-10-27

**Authors:** Chao-Chi Huang, Yang-Hung Chiu, Chih-Yu Wen

**Affiliations:** Department of Electrical Engineering, Graduate Institute of Communication Engineering, National Chung Hsing University, Taichung 402, Taiwan; E-Mails: k-122334455@hotmail.com (C.-C.H.); ace19622001@hotmail.com (Y.-H.C.)

**Keywords:** telematics, vehicular sensor networks, topology control

## Abstract

In a vehicular sensor network (VSN), the key design issue is how to organize vehicles effectively, such that the local network topology can be stabilized quickly. In this work, each vehicle with on-board sensors can be considered as a local controller associated with a group of communication members. In order to balance the load among the nodes and govern the local topology change, a group formation scheme using localized criteria is implemented. The proposed distributed topology control method focuses on reducing the rate of group member change and avoiding the unnecessary information exchange. Two major phases are sequentially applied to choose the group members of each vehicle using hybrid angle/distance information. The operation of Phase I is based on the concept of the cone-based method, which can select the desired vehicles quickly. Afterwards, the proposed time-slot method is further applied to stabilize the network topology. Given the network structure in Phase I, a routing scheme is presented in Phase II. The network behaviors are explored through simulation and analysis in a variety of scenarios. The results show that the proposed mechanism is a scalable and effective control framework for VSNs.

## 1. Introduction

Vehicular sensor networks (VSNs) are emerging as a new solution for inter-vehicle communication or monitoring urban environments, such as intelligent transportation systems [1]. Recently, many protocols for *ad hoc* networks have been proposed with the advances in wireless network communications, especially those based on the mobile *ad hoc* network (MANET) architecture derived from the vehicular *ad hoc* network (VANET). Although some of the existing *ad hoc* and sensor network communication protocols can still be applied to vehicular networks (both VSNs and VANETs), simulation results [2,3] have showed that they suffer from poor performances, because of the fast movements of vehicles and limited chances for information exchanges. Furthermore, without a robust infrastructure, nodes (or vehicles) in an *ad hoc* network may be required to self-organize. Therefore, the design principles for guaranteeing basic performance achievement in an *ad hoc* network is to make an *ad hoc* network more stable and further provide scalability and robustness for data dissemination in the network [4].

**Figure 1.**
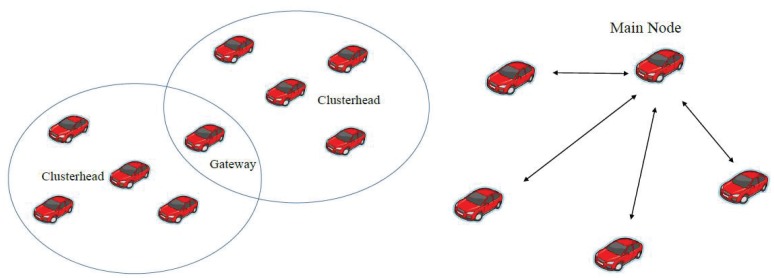
Cluster-based scheme (**Left**); distributed scheme (**Right**).

In general, two kinds of topology control are considered: (1) cluster-based topology control [5–7]; and (2) distributed topology control [8,9] (Figure 1). In cluster-based topology control, maintaining network connectivity is crucial. An implementation of the linked cluster architecture may consider the following tasks: cluster formation, cluster connectivity and cluster reorganization. However, it suffers from network partitions, which are very common in vehicular networks and may lead to poor system performances, due to a highly dynamic network topology and limited and unstable network resources for communication [10]. In distributed topology control, the main node may use network characteristics and a mobility model to find its group members (e.g., distance, driving direction or speed) for communication and information routing. The conceptual design principles for topology control in VANETs can be found in [11].

In this work, we focus on distributed topology control for achieving effective topology management in a vehicle sensor network. In this category, two major ways to govern mobile nodes are the cone-based scheme [8] and the distance scheme [9]. However, these two schemes may not be effective with regards to node management in the scenarios with changeable moving speeds and directions (e.g., turning left/right). Accordingly, we combine the concepts of these two schemes and propose a hybrid scheme, the hybrid distributed topology control scheme (HDTC), using angle and distance information to form a communication group for each vehicle, which relies on a distributed self-configuring protocol [12]. On the basis of the corresponding communication group, a routing group member is further selected to forward the information to the sink vehicle.

The key features and contributions of the HDTC are as follows: (1) a distributed topology control scheme is proposed to select the communication members that will participate information exchange; (2) the proposed HDTC scheme combines the strength of the cone-based scheme [8] and the distance scheme [9] to achieve an adaptive topology management for VSNs; (3) extensive experiments are carried out to evaluate HDTC with several scenarios. Our experiment results show that the proposed HDTC algorithm can effectively perform network topology management. Compared with the cone-based scheme and the distance scheme, the proposed HDTC has lower communication complexity and provides a more stable communication network structure for information routing.

This paper is organized as follows: Section 2 reviews related works about the topology control problem and information dissemination protocols. Section 3 describes the approach of the HDTC scheme for forming the communication group and determining the routing group members. In order to facilitate performance evaluation of a protocol design, Section 4 presents an analytical tool to estimate the number of communication group members, and Section 5 depicts the frequency of topology change and the link up/down dynamics. In Section 6, we evaluate the system performance. Finally, Section 7 draws conclusions and shows future research directions.

## 2. Literature Review

There are two primary related research areas: (1) the topology control problem; and (2) information dissemination protocols. For the topology control problem, there are two major categories for the topology control solutions: Cluster-based topology control and distributed topology control. A large variety of approaches for *ad hoc* clustering have been proposed in the literature [13–18]. Most of these design approaches are heuristic protocols in which each sensor must maintain knowledge of the complete network or identify a subset of sensors with a clusterhead to partition the network into clusters in heuristic ways.

The authors in [13] present a novel cluster-based network topology discovery approach for VANETs by applying a cluster formation procedure similar to the one used in the max-min *d*-hop heuristic approach [14] and utilizes the advantage of a *d*-hop cluster architecture to improve the network topology scalability. However, clusters are formed heuristically without taking the cluster size and their mobility pattern into consideration. Vodopivec *et al.* [15] propose a new clustering metric and a clustering algorithm with multi-homing support. It relies only on the vehicle's ability to send and receive wireless packets, which identify the vehicle relationship. Clusters are created with redundant connections between nodes to increase the communication reliability in case of topological changes. Nonetheless, this protocol design leads to high communication overheads. In [16], a clustering technique suitable for the VANET environment on highways is proposed for enhancing the stability of the network topology. This technique takes the speed difference as a parameter to create a relatively stable cluster structure. Note that the traffic pattern is limited to the roads with an uninterrupted flow of traffic. Our previous work [17] focuses on cluster-based topology management of nodes with “low” or “moderate” mobility. The mobile nodes use random timers to form clusters, including cluster heads, cluster members and gateways, in a distributed way. However, the above control scheme may not work for the nodes with high mobility (e.g., vehicles) in a highly dynamic topology.

For distributed topology control, the authors in [8] propose a cone-based distributed topology-control (CBTC) algorithm with directional information and without GPS information. The authors show that taking the cone angle *α* = 5/6*π* is a necessary and sufficient condition to guarantee that network connectivity is preserved. Even though a set of refinements are further proposed to reduce power consumption and prove that they retain network connectivity, dynamic reconfiguration and complex optimizations are required to maintain the network operations. In [9], the authors utilize the geometric model in which the threshold is determined by the Euclidean distance *D_uυ_* between vehicle *u* and vehicle *ν*. The threshold is taken to be *D_uυ_* · *α*, where *α* is the attenuation constant associated with path loss [19]. It is assumed that each node moves at its own constant rate and direction throughout the time interval, such that the lifetime of a mobile network may be sliced into unit time intervals. Accordingly, the topology control problem for each unit time interval is investigated. Although the network is movement-connected throughout the unit time interval, the implementation complexity for solving the topology stability problem is high. Comprehensive surveys of recently proposed topology control algorithms for mobile *ad hoc* and sensor networks can be found in [20–22].

For the information dissemination problem, several VSN routing protocols have been proposed for inter-vehicle communication in recent years. Topology-based and position-based routing are two possible strategies of data forwarding commonly adopted for multi-hop wireless networks [23,24]. Topology-based protocols use the information of available network links for packet transmission. Position-based protocols assume that every node is aware of the location of itself, the location of neighboring nodes and the location of the destination node. With the increasing availability of GPS-equipped vehicles, the position-based protocols are getting more convenient. However, the position-based protocols developed for MANETs may not directly be applied to vehicular environments, due to the unique vehicular network characteristics. There are a number of papers that study packet routing algorithms in VSNs [25–31]. With the use of geographical location information obtained from GPS devices, studies show that position-based routing (geographic routing) has been identified as a more promising routing paradigm for vehicles [29,30]. Nonetheless, most existing position-based or topology-based VANET/VSN routing protocols assume that intermediate nodes can be found to set up an end-to-end connection; otherwise, the data packet will be dropped by the protocols. However, finding end-to-end connections sometimes is extremely difficult for a sparse vehicular network. Under this circumstance, topology control schemes may be applied to provide basic levels of system performance.

In contrast, the proposed self-organization strategy (HDTC) investigates the design of topology management and information dissemination and considers several important factors, such as the contribution of vehicle mobility, a sensor vehicle joining/leaving a communication group and the routing member selection, in order to keep the dynamic network structure efficient. It applies localized criteria and maintains local dynamic topologies in a fully distributed way. Therefore, the proposed HDTC scheme combines the strength of topology control and the one-hop information exchange between neighboring vehicles to achieve sufficient network reachability under high mobility environments. The comparison of the proposed scheme and other control-based approaches [8,9] is further discussed in Section 6.

## 3. The Hybrid Distributed Topology Control Scheme (HDTC)

**Figure 2.**
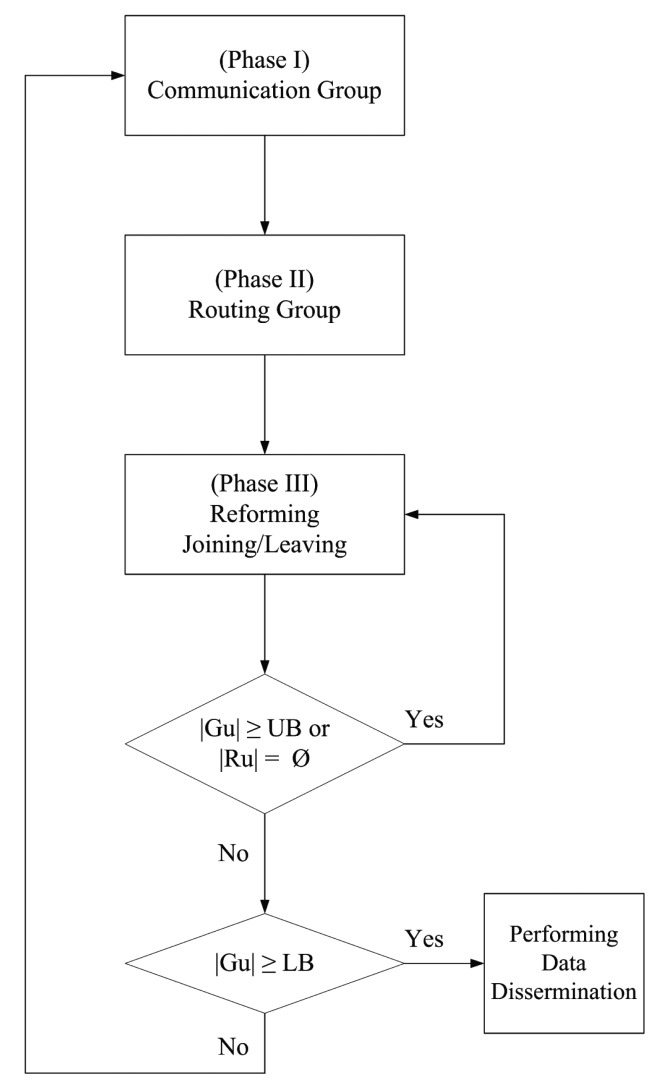
Flow diagram of the hybrid distributed topology control (HDTC) scheme. UB, upper bound; LB, lower bound.

This section presents an inter-vehicle topology control protocol, HDTC, which combines the concept of the cone-based scheme [8] and the proposed time-slot scheme to govern a group of vehicles in a vehicular sensor network and to decide the manner of data exchange among vehicles with on-board sensors. The proposed HDTC consists of three phases. In Phase I, the modified cone-based scheme and the time-slot scheme are proposed for the initial selection of group members. Afterwards, a hybrid solution is introduced for selection refinement and forming a communication group. Note that the transmission range of a sensor defines the communication area in which it can interact with other sensor devices. On the basis of Phase I, a distributed routing scheme is developed in Phase II. In order to keep the local network structure stable, in Phase III, a scheme for reforming the communication/routing group is presented. Figure 2 shows the conceptual flow diagram of the HDTC scheme, where *G_u_* and *R_u_* are the sets of communication/routing groups for vehicle *u* and UB and LB are the upper and lower bounds of |*G_u_*|, respectively. Note that the function of the upper and lower bounds of |*G_u_*| is to maintain the local communication structure, especially for a sparse network.

The main assumptions are: (1) all sensors are homogeneous with the same transmission range; (2) the sensors have sensing and communication capability; (3) the position information of the source and destination vehicles is known; (4) the trajectory characteristics of the destination vehicle are known; and (5) periodic messages are transmitted at regular intervals to inform the neighboring vehicles. Note that there are no base stations to coordinate or supervise activities among sensors/vehicles.

### 3.1. Phase I: Forming the Communication Group

#### 3.1.1. Initial Selection (Group 1): Modified Cone-Based Method

**Figure 3.**
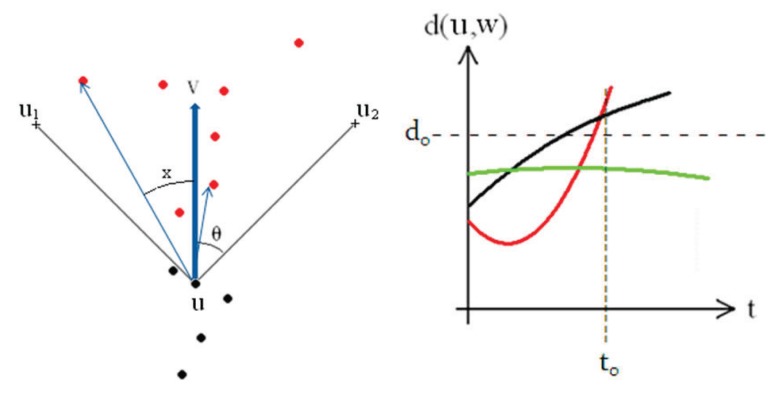
The cone-based scheme (**Left**); the time-slot scheme (**Right**).

In order to determine the initial forwarding direction, we may apply the geometrical information and the moving directions of the source and destination vehicles. Referring to Figure 3, define the forward cone:

(1) $$K_{+}=\{\upsilon_{w}\in R^{2}:\cos(\theta )<(\upsilon_{w},\upsilon_{u})<1\}$$

where *υ*(·) is the normalized unit vector of movement, (·, ·) denotes the ordinary inner product on ℛ^2^, *θ* is related to the opening angle of the cone and 0 < cos(*θ*) < 1. Analogously, the backward cone *K*_−_ is defined by the property:

(2) $$K_{-}=\{\upsilon_{w}\in R^{2}:-1<(\upsilon_{w},\upsilon_{u})<-\cos(\theta )\}$$

For a fixed destination vehicle, the forwarding cone can be determined by:

- If the inner product ( $\vec{P_{u}P_{w}}$, *υ_u_*) ≥ 0, forward the information based on *υ_u_*.
- If the inner product ( $\vec{P_{u}P_{w}}$, *υ_u_*) < 0, forward the information based on −*υ_u_*.

Note that *P_u_* is the position information of vehicle *u*. Similarly, for a moving destination vehicle, the criteria are:

- If (*υ_w_*, *υ_u_*) ≥ 0 & ( $\vec{P_{u}P_{w}}$, *υ_u_*), ≥ 0, forward the information based on *υ_u_*.
- If (*υ_w_*, *υ_u_*) ≥ 0 & ( $\vec{P_{u}P_{w}}$, *υ_u_*) < 0, forward the information based on −*υ_u_*.
- If (*υ_w_, υ_u_*) < 0, select the nearest vehicle in the opposite moving direction. Then follow the above two steps.

**Figure 4.**
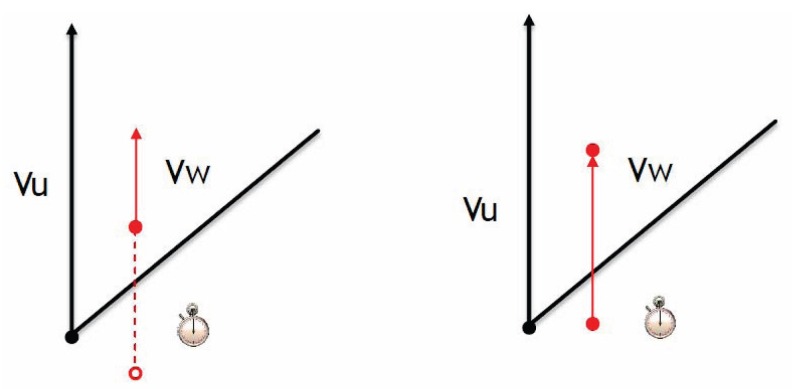
The timer implementation in the modified cone-based scheme: Vehicle *w* moves slower than vehicle *u* (**Left**); vehicle *w* moves faster than vehicle *u* (**Right**).

As shown in Figure 3 (left), in order to form the communication group of vehicle *u*, let the forward vector *υ_u_* be a reference direction. Note that the forward vector *υ*(·) can be determined by the geometrical information of the destination vehicle. Based on the vector *υ_u_*, vehicles are selected to be group members of *u*. The cone of degree 2*θ* is bisected by the vector *υ_u_*. Referring to Figure 3 (left), if a node has the angle information 0 ≤ cos *X* ≤ cos*θ*, add it to Group 1; otherwise, it is excluded. In the modified cone-based method, when the node moves slower than the observation node (say, vehicle *u*), there may be a gap to make the role of a node oscillate between being a group member and not being a group member. Thus, as shown in Figure 4, in order to reduce the occurrence frequency of this scenario, a timer is implemented in the cone-based scheme to further observe the behaviors of neighboring vehicles. Consequently, we renew the timer as long as the angle information of a node passes the test of the cone-based method.

#### 3.1.2. Initial Selection (Group 2): Proposed Time-Slot Method

Based on the distance measurement and directional information of its neighboring vehicle *w*, vehicle *u* can project whether, after *t*_0_ seconds, the neighboring vehicle *w* may be *d*_0_ meters away or not. If not, add the node *w* to Group 2; otherwise, it is excluded. Figure 3 (right) shows the distance between the vehicle *u* and the vehicle *w*. The distance information at time *t*_0_ is applied to select the group members. Observe that the vehicle *w* may be away from *u*, because it is faster than *u* (just like the black line), or as described in the red line, *u* is faster, so that *u* overtakes *w*. In contrast, we prefer the vehicle *w* steadily close to vehicle *u*, as depicted in the green line.

#### 3.1.3. Hybrid Scheme

**Figure 5.**
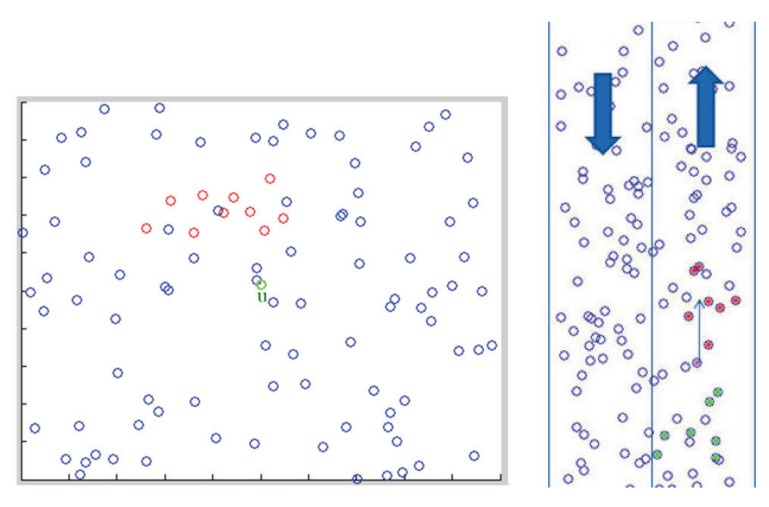
The proposed HDTC scheme (**Left**); the vehicle and its group members (**Right**).

The vehicle *u* (green node) uses the above procedures to find the nodes belonging to Group 1 and Group 2. As shown in Figure 5 (left), the nodes (red nodes), which are marked in both Group 1 and Group 2, are candidates of the group members. Therefore, we have:

(3) $$G_{1}=\{p^{\prime }|p^{\prime }\in N_{u},\cos X_{\left(p^{\prime }\right)}\leq \cos\theta \}$$

(4) $$G_{2}=\{p^{\prime }|p^{\prime }\in N_{u},D_{up^{\prime }}(t_{0})\leq d_{0}\}$$

(5) $$G_{u}=\{p^{\prime }|p^{\prime }\in (N_{u}\cap G_{1}\cap G_{2}\}$$

where *N_u_* is the set of neighboring vehicles of vehicle *u. D_up′_* is the distance between vehicle *p′* and vehicle *u.* Accordingly, the vehicle *u* sends a message “join” to its neighboring member nodes. When the nodes, which belong to the set *G_u_*, receive this message, they join the group of vehicle *u* and form the communication group of vehicle *u.*

**Figure 6.**
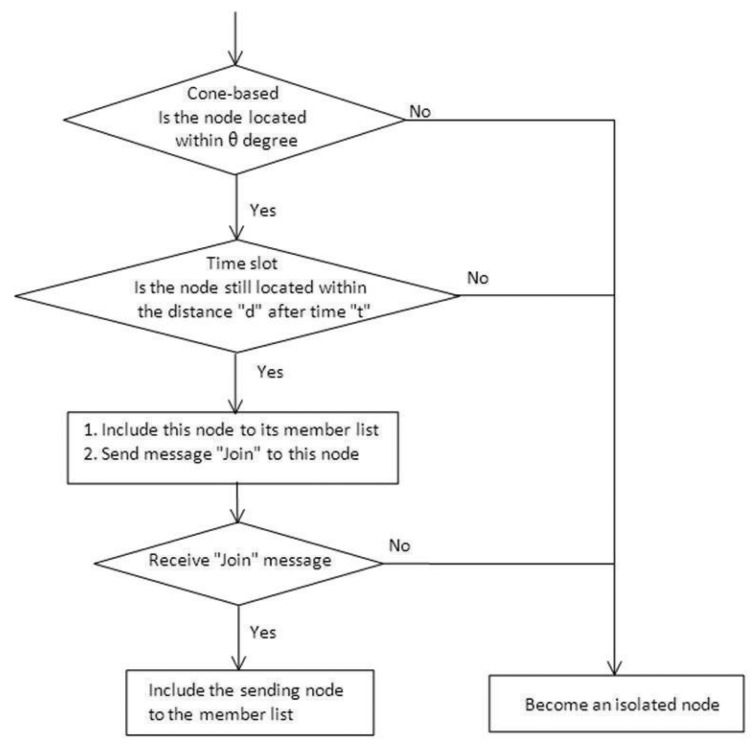
Flow diagram of the HDTC scheme.

Figure 5 (right) shows a traffic scenario. The right-hand side is an up vector for representing the moving direction of vehicles, and the left-hand side is a down vector. Note that the purple node is the observation node (say, node *u*), and red nodes are the members that are found by the purple node after performing the proposed method. Figure 6 depicts the flow diagram of the proposed scheme. First, we use the cone-based method to check the nodes. Secondly, we use the time-slot scheme to test the distance information. Third, group those nodes into members, and send the message to them. Finally, when receiving a message from other nodes, the node becomes a group member associated with the message with the earliest time stamp.

### 3.2. Phase II: Determining the Routing Group Members

In order to determine the routing group member, a priority function may be implemented based on the communication group in Phase I. From the routing group, a member is selected to perform the information relay (e.g., the nearest one with respect to the sink node). Accordingly, each node has its own routing group members and the relay vehicle. Thus, we obtain:

(6) $$R_{u}=\{p^{\prime }|p^{\prime }\in G_{u},\cos Y_{\left(p^{\prime }\right)}\leq \cos\theta^{\prime }\}$$

(7) $$\begin{array}{cccc} p^{\prime }= & \arg & \min & D_{p^{\prime }\text{sink}}\\ & & p^{\prime }\in R_{u} & \end{array}$$

where *D_p′sink_* is the distance between vehicle *p′* and the sink vehicle, *θ′* = *η* · *θ* and 0 < *η* ≤ 1. The rationale of the parameter *θ′* is to take the group moving characteristics into account, such that proper candidates for forwarding the message to the sink vehicle can be determined. Note that the position of vehicle *p′* can be estimated by the angle/distance information associated with the position of vehicle *u*. Therefore, vehicle *p′* is selected as the relay node for vehicle *u.*

**Figure 7.**
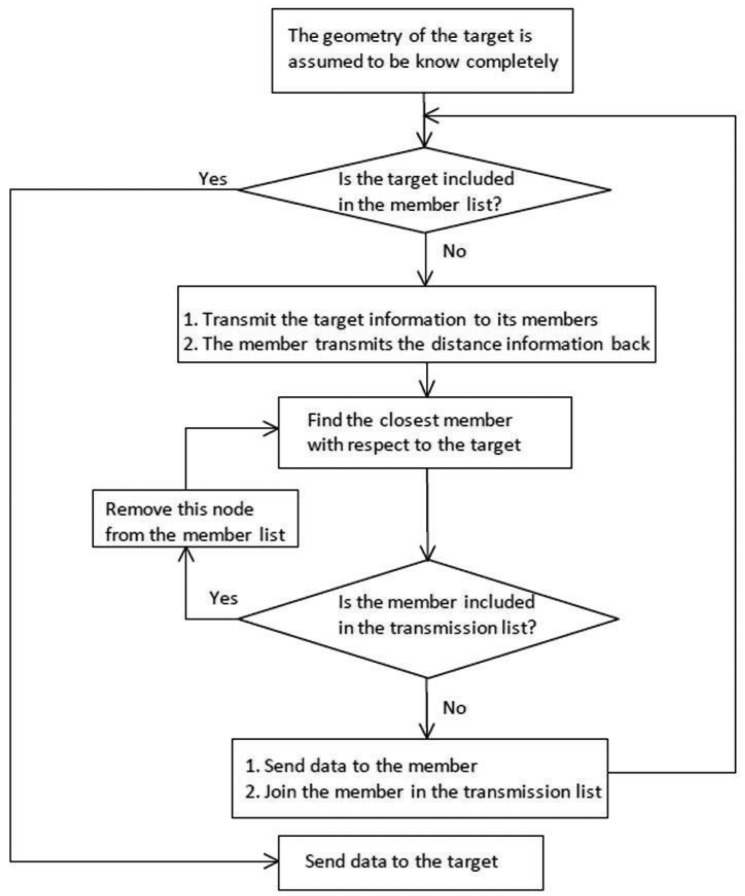
Flow diagram of Phase II: Determining the routing group members.

**Figure 8.**
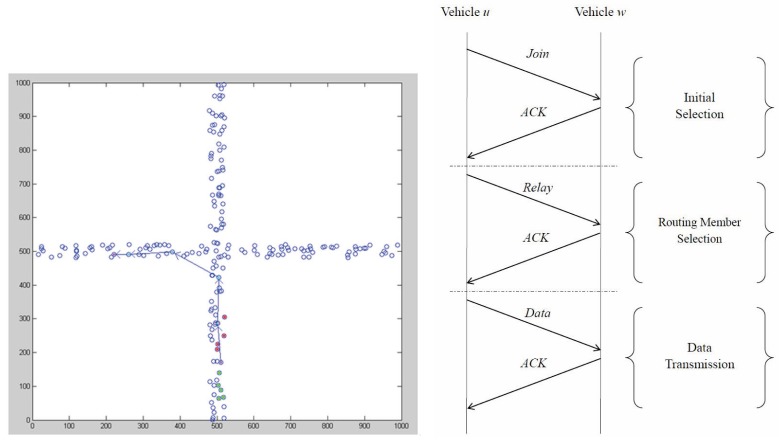
A typical example of the proposed HDTC scheme for information routing (**Left**); one-hop communication protocol (**Right**).

Figure 7 depicts the flow graph of Phase II, and Figure 8 (right) demonstrates the communication protocol, including initial selection, routing member selection and data dissemination. Figure 8 (left) shows an example for the proposed algorithm in an intersection. The purple node at (510, 150) is the source node, which is going up, and the second purple node at (200, 490) is the sink node, which is going right. The ocean blue nodes are the relay nodes.

### 3.3. Phase III: Reforming the Communication/Routing Group

The proposed group reformation strategy considers a vehicle joining/leaving a group and maintains a group in a fully-distributed way. In order to keep the local network structure stable, the group size may be a key parameter to achieve sufficient network reachability. However, due to node movement, the group size changes as time proceeds. In the proposed approach, referring to the neighboring information and its group size, each vehicle (e.g., vehicle *u*) triggers its group update process if necessary. The following subsections describe possible schemes for handling new admissions and releases of nodes in a group.

#### 3.3.1. Communication Group *G_u_*

Let the upper bound *UB*and the lower bound *LB*represent the constraints of the group size for managing the topology change. When the group size is over the upper bound *UB* (*i.e.*, |*G_u_*| ≥ *UB*), update *G_u_* and *R_u_*; while *LB* ≤ |*G_u_*| < *UB*, monitor and update *R_u_* if necessary; when the group size is under the lower bound *LB*, reform the communication group and go to Phase I. Thus, each group size is adjusted autonomously.

Accordingly, vehicle *u* may include its new neighbors when the group size constraint is satisfied. On the other hand, when a vehicle leaves a group, this link down event can be detected by not receiving periodical broadcasting messages, and the neighboring vehicles can update the knowledge of their neighborhood. This phase performs the group reformation, which aims to maintain topology stability. The reformation conditions are:

(1) If |*G_u_*| ≥ *UB*, update *G_u_* and *R_u_*.
(2) If *UB* > |*G_u_*| ≥ *LB*, go to Phase II, monitoring and updating the routing group members *R_u_*.
(3) If |*G_u_*| < *LB*, increase *θ* and go to Phase I (*i.e.*, reforming *G_u_*).

Notice that since the neighbor vehicle properties depends on the type of mobility occurring in the network, the upper bound *UB* and the lower bound *LB* of the group size are given according to the number of vehicles and issues on the mobility model.

#### 3.3.2. Relay Reselection

Due to the vehicle movements, adaptive reselection of the relay member is necessary for information dissemination. Therefore, one simple reselection strategy is described as follows: When *R_u_* is empty, we have:

(8) $$\begin{array}{cccc} p^{\prime }= & \arg & \min & D_{p^{\prime }\text{sink}}\\ & & p^{\prime }\in G_{u} & \end{array}$$

where the relay member is selected from *G_u_*. Otherwise, the relay vehicle is selected by:

(9) $$\begin{array}{cccc} p^{\prime }= & \arg & \min & D_{p^{\prime }\text{sink}}\\ & & p^{\prime }\in R_{u} & \end{array}$$

where the relay member is selected from *R_u_*. The procedures of the HDTC scheme and information routing are summarized in Table 1.

## 4. Probabilistic Model (PM) for the Distributed Approach

In order to abstract the network behavior and estimate the number of communication group members, here, we present a probabilistic model (PM) for vehicles with random movements. Here, the concepts of the similarity measure and flexibility measure are introduced to assign a probability of a vehicle for being a communication group member. On the basis of probability assignment, the Lindeberg theorem is further applied to estimate the communication group size. Readers are referred to [32] for a complete discussion and proof of the theorem.

### 4.1. Similarity and Flexibility Measures

**Figure 9.**
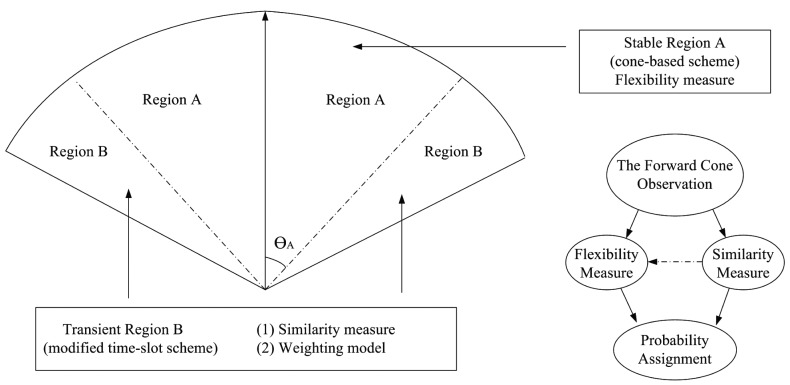
The probabilistic model of the proposed HDTC scheme.

Considering the angle *θ* between *υ_u_* and *υ_w_*, as depicted in Figure 3 (left), the information of moving direction may be further used to weight the possibility for being a communication group member. In order to weight the importance of each neighboring vehicle, the forward cone is divided into two regions, A and B. As depicted in Figure 9, let Region A and Region B be the cone with the opening angle 2*θ_A_*(*θ_A_* ≤ *θ*) and the transition transient area, respectively Note that the parameter *θ_A_* (*i.e.*, half of the opening angle of Region A) can be regarded as a measure for the “flexibility” of neighboring vehicles with respect to the source vehicle *u.* Thus, under the circumstance of a high moving speed, a small value of *θ_A_* may be applied. Accordingly, for the vehicles inside Region A, the probability for being a group member is given by:

(10) $$p_{w}=1,\forall w\in A$$

For the vehicles inside Region B, the concept of the “similarity” measure and the weighting model of moving direction between vehicles *w* and *u* are used to determine the probability for being a group member, which yields:

(11) $$p_{w}=\{\begin{array}{ll} (\upsilon_{w},\upsilon_{u}), & \text{if}(\upsilon_{w},\upsilon_{u})>0\\ \gamma \cdot |(\upsilon_{w},\upsilon_{u})|, & \text{otherwise} \end{array}$$

where 0 < *γ* < 1 and (*υ_w_, υ_u_*) is the ordinary inner product for the moving vectors of vehicles *w* and *u*.

Note that the above design logic is attributed to the fact that Region A is considered as the major area for determining the group members. That is, the vehicles in Region A may have a larger chance to be selected as a communication group member. In contrast, Region B is the area, where the frequency of the link up/down of the neighboring vehicles with respect to vehicle *u* may be high. Thus, in Region B, the probability for being a group member *p_w_* is adjusted by a “similarity” measure. Therefore, the probability assignment of the neighboring vehicles for being the communication group members of the source vehicle *u* is complete.

### 4.2. Lindeberg Theorem

Suppose for each *n*,

$$\left(X_{n1},X_{n2},\ldots ,X_{nr_{n}}\right)$$

is an independent random vector. The probability space may change with *n*, and the set of these vectors is called a triangular array of random variables. Put *S_n_* = *X_n_*_1_ + ⋯ + *X_nr_n__*. In the network application, let *X_ni_* be *X_i_*, and let *X_i_* take the values one and zero with probability *p_i_* and *q_i_* = 1 − *p_i_*, respectively. We may interpret *X_i_* as an indicator that sensor *i* is chosen to be a clusterhead with probability *p_i_* and *S_n_* is the number of clusterheads in a network.

Denote *Y_i_* = *X_i_* − *p_i_*. Hence,

(12) $$S_{n}^{Y}=\sum_{i=1}^{n}Y_{i}=S_{n}-\sum_{i=1}^{n}p_{i}$$

with *E*[*Y_i_*] = 0, $\sigma_{Y_{i}}^{2}=\sigma_{X_{i}}^{2}=p_{i}(1-p_{i})$, and $\sigma_{s_{n}}^{2}=\sum_{i=1}^{n}\sigma_{Y_{i}}^{2}=\sum_{i=1}^{n}\sigma_{X_{i}}^{2}$. For our case, the Lindeberg condition [32] reduces to:

(13) $$\begin{array}{c} \lim\\ n\to \infty \end{array}\sum_{i=1}^{n}\frac{1}{s_{n}^{2}}\int_{|Y_{i}|\geq \epsilon s_{n}}{Y_{i}}^{2}dp\leq \begin{array}{c} \lim\\ n\to \infty \end{array}\sum_{i=1}^{n}\frac{1}{s_{n}^{2}}\int_{|Y_{i}|\geq \epsilon s_{n}}dp=0$$

which holds, because all the random variables are bounded by one and [|*Y_i_*| ≥ ∊*s_n_*] → 0 as *n* → ∞.

#### Theorem 1.

*Suppose that Y_i_* *is an independent sequence of random variables and satisfies E*[*Y_i_*] = 0, $\sigma_{Y_{i}}^{2}=E[Y_{i}^{2}]$, $S_{n}^{Y}=\sum_{i=1}^{n}Y_{i}$ *and* $s_{n}^{2}=\sum_{i=1}^{n}\sigma_{Y_{i}}^{2}$. *If the Lindeberg condition (13) holds, then* $S_{n}^{Y}/s_{n}\to N(0,1)$.

Consequently, referring to Equations (10) and (11), and Theorem 1, the distribution of the number of group members of vehicle *u* can be approximated by $N(\mu_{u},\sigma_{u}^{2})$ with $\mu_{u}=\sum_{i=1}^{m_{u}}p_{i}^{\left(u\right)}$ and $\sigma_{u}^{2}=\sum_{i=1}^{m_{u}}p_{i}^{\left(u\right)}(1-p_{i}^{\left(u\right)})$, where $p_{i}^{\left(u\right)}$is the probability for being a group member of vehicle *i* with respect to vehicle *u.*

## 5. Analysis of Link Up/Down Dynamics

This sections presents the link available time distribution of a one-hop connectivity between two vehicles, which is applied to analyze the link up/down dynamics in a communication group.

### 5.1. Link Available Time

Assume the initial distance between the source vehicle and a group member is *r* and consider two mobile nodes, a source vehicle and a group member, with the velocity vectors **v**_1_ and **v**_2_, respectively, which implies that the relative velocity vector is **v***_r_* = **v**_1_ − **v**_2_ at a given time *t*. After time Δ*t*, a group member may move out of the transmission range of the source vehicle. Referring to the derivation in [33], the probability that the link available time is less than Δ*t* is given by:

(14) $$P_{lat}(r,\psi ;\Delta t)=\int_{0}^{2\pi }\int_{\upsilon_{\mu }}^{\infty }f_{\upsilon_{r}}(\upsilon_{r},\theta_{r})d\upsilon_{r}d\theta_{r}$$

where *ψ* is an angle between the one-hop link between a source vehicle and a group member, the X-axis, *υ_μ_* = *μ*(*r, θ_r_*)/Δ*t, f_υ_*(*υ_r_, θ_r_*) is the polar form of the relative velocity probability density function and *μ*(*r, θ_r_*) is a function of the initial distance *r* between the source vehicle and a group member and the angle *θ_r_* between the relative moving direction of the group member and the line connecting these two vehicles. Therefore, through the averaged analysis, the mean link available time distribution between two arbitrarily chosen vehicles yields:

(15) $${\bar{P}}_{lat}(\Delta t)=\int_{0}^{2\pi }\int_{0}^{R_{M}}P_{lat}(r,\psi ;\Delta t)rdrd\psi$$

which is the averaged probability that the link is available for less than Δ*t* seconds. The frequency of topology change and the link up/down dynamics is further discussed and illustrated via simulation in Section 6.

### 5.2. Analysis of Density Change

To advance the investigation, let *f_Du_*(*t*) be the node density in the communication group of vehicle *u* and $N_{\text{in}}^{\left(u\right)}(t)$ and $N_{\text{out}}^{\left(u\right)}(t)$ represent the number of nodes moving in and out of group *u* at time *t*, respectively. Therefore, at time *t* + Δ*t*, in group *u*, the node density change Δ*f_D_u__*(*t* + Δ*t*) can be expressed by:

(16) $$\Delta f_{D_{u}}(t+\Delta t)=\frac{N_{\text{in}}^{\left(u\right)}(t+\Delta t)-N_{\text{out}}^{\left(u\right)}(t+\Delta t)}{A_{u}}$$

where *A_u_* is the area size of group *u*. According to the criteria for reforming the communication/routing group (as described in Section 3.3), the relationships between the node density change and the operation of group reformation are:

(1) If |*G_u_*| ≥ *UB*, update *G_u_* and *R_u_*.
  (17) $$\int_{t_{0}}^{t_{1}}\Delta f_{D_{u}}(\tau )d\tau >\frac{UB-N_{GM}^{\left(u\right)}(t_{0})}{A_{u}}$$
(2) If *UB* > |*G_u_*| ≥ *LB*, go to Phase II, monitoring and updating the routing group members *R_u_*.
  (18) $$\frac{LB-N_{GM}^{\left(u\right)}(t_{0})}{A_{u}}<\int_{t_{0}}^{t_{1}}\Delta f_{D_{u}}(\tau )d\tau <\frac{UB-N_{GM}^{\left(u\right)}(t_{0})}{A_{u}}$$
(3) If |*G_u_*| < *LB*, increase *θ* and go to Phase I (*i.e.*, reforming *G_u_*).
  (19) $$\int_{t_{0}}^{t_{1}}\Delta f_{D_{u}}(\tau )d\tau <\frac{LB-N_{GM}^{\left(u\right)}(t_{0})}{A_{u}}$$
  where $N_{GM}^{\left(u\right)}(t_{0})$ is the number of group members in group *u* at time *t*_0_ and *t*_1_ is the time stamp to trigger the process of group reformation (*t*_1_ > *t*_0_). Accordingly, the above criteria can be applied to perform group reformation.

## 6. Simulation Results

This section introduces the traffic environment and provides performance analysis via MATLAB simulations. Several arguments (e.g., the cone angle *θ*, transmission range and the speed of vehicle) are explored to observe network behaviors in several different environments. Here, we compare the performance of forming the communication group and information routing with three schemes, including: (1) the distance method [9]; (2) the cone-based method [8]; and (3) the proposed HDTC method. The relationship between the arguments and the communication group and the relationship between the arguments and the routing group are discussed, respectively. The default settings in the simulation are: *θ* = 75° (*i.e.*, half of the cone angle); the average speed is 21 m/s; the connection distance is 150 m. Let *θ′* ≈ (2/3)*θ* for the purpose of selecting the routing group. In [34], a long-range radio is applied for the sensors in outdoor applications, and the experimental results show that the average communication range is about 200 m. Accordingly, the proposed scheme is examined with varying transmission ranges from 110∼200 m. In the proposed system, the short timer of the modified cone-based scheme is set to be 4 s and the threshold distance *d*_0_ of the time-slot method is set to be the transmission range. The default lane headway is assumed to be 20 m. Assume the upper and lower bounds of |*G_u_*| are UB = 20 and LB = 5. Table 2 depicts the values of simulation parameters.

### 6.1. Experimental Environments

**Figure 10.**
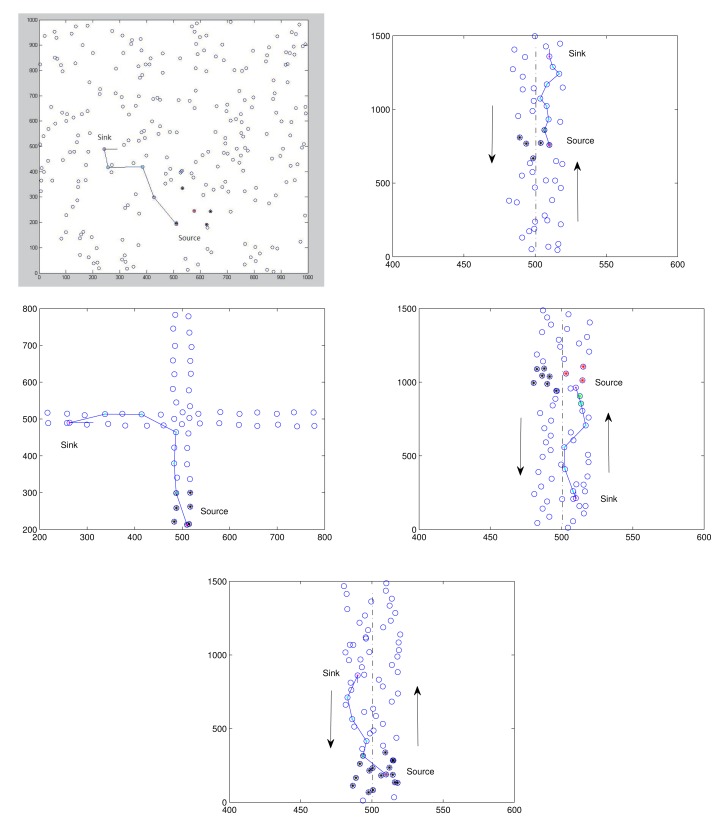
Examples of the HDTC scheme and information routing: Scenario 1 (**Top Left**); Scenario 2, forward propagation (**Top Right**); Scenario 3 (**Middle Left**); Scenario 4, backward propagation (**Middle Right**); and Scenario 5 (**Bottom**).

As illustrated in Figure 10, three different traffic scenarios and two special cases are considered to examine the system performance: (1) Scenario 1, a random walk; (2) Scenario 2, a straight-line moving; (3) Scenario 3, moving through an intersection; (4) Scenario 4, a backward propagation; and (5) Scenario 5, the sink and the source vehicles traveling in the opposite direction. The arrows represent the moving directions. For Scenario 1 (Figure 10 (top left)), associated with each moving node is a random position. It is assumed that each node moves at its own uniform rate and direction. The source node stars from (510, 150) to move up; the sink node stars from (200, 490) to move right. For Scenario 2, Figure 10 (top right) shows a straight-line moving model on a straight 40 meter-wide road. For Scenario 3, Figure 10 (middle left) shows a moving model, where the nodes are traveling through an intersection. The source node and the sink node move up and move forward to the right, respectively. Compared with Scenario 2, two special cases (Scenarios 4 (Figure 10 (middle right)) and 5 (Figure 10 (bottom))) have similar system performances. That is, for a backward propagation case (*i.e*., the sink vehicle is behind the source vehicle) and for the case that the sink and the source vehicles are traveling in the opposite direction, the network behaviors are close to those of Scenario 2. In the following subsections, we will focus on the performance discussion of Scenarios 1∼3.

### 6.2. Performance Evaluation

Referring to the settings in Section 6.1, this subsection investigates the performance with regard to the group size, the number of in/out group members (*i.e*., the link up/down dynamics), the connection time and the number of hops for information routing, with varying *θ*, the transmission range and the vehicle speed, respectively.

#### 6.2.1. Scenario 1

**Figure 11.**
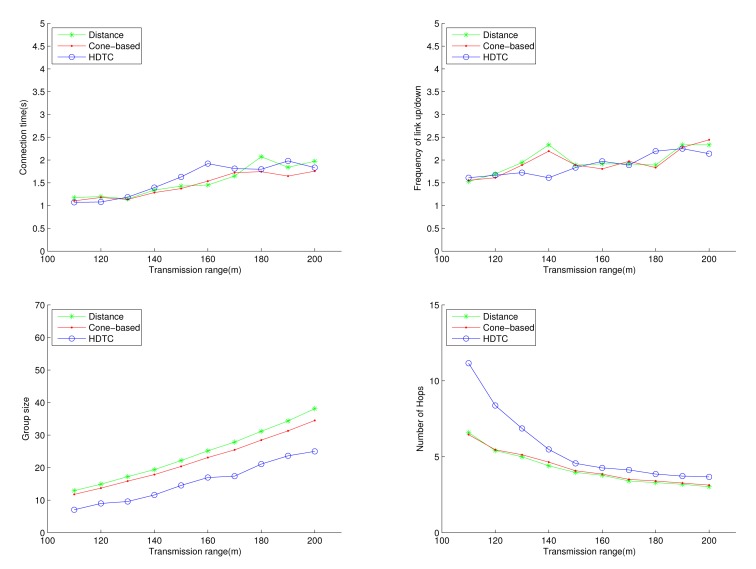
Scenario 1: The connection time, the frequency of the link up/down, the group size and the number of hops with varying the connection distance.

**Figure 12.**
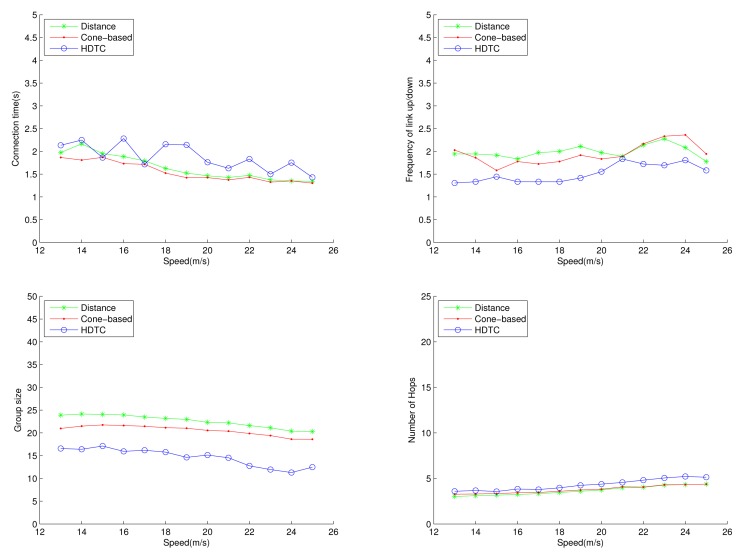
Scenario 1: The connection time, the frequency of the link up/down, the group size and the number of hops with varying the vehicle speed.

**Figure 13.**
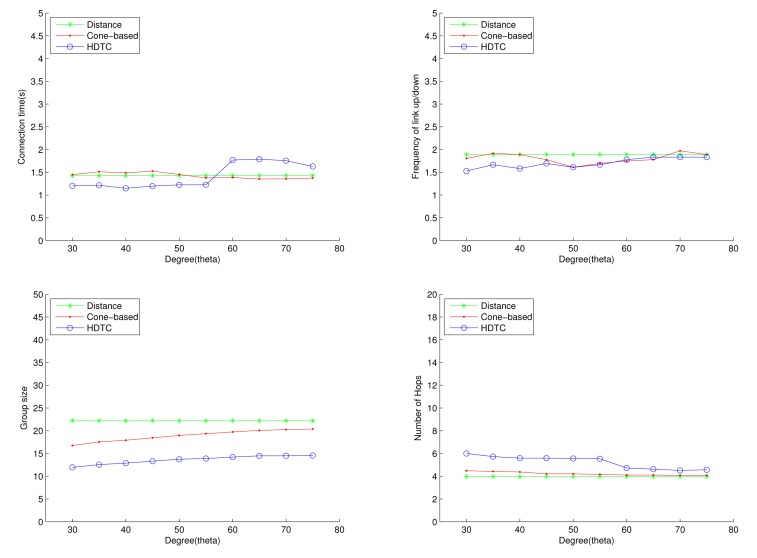
Scenario 1: The connection time, the frequency of the link up/down, the group size and the number of hops with varying *θ*.

Under the circumstances of random movement of the vehicles, Figures 11, 12 and 13 depict the network behaviors, considering the transmission range, the vehicle speed and the cone angle, respectively. Assume that 250 nodes are deployed in the network. Figure 11 (bottom left) shows that the group size of HDTC is smaller than those of the distance method [9] and the cone-based method [8]. This is because the proposed time-slot method filters out the vehicle nodes that move away from the source/relay vehicle. Moreover, referring to Figure 11 (bottom right), a smaller transmission range (e.g., 110 m) leads to a smaller group size, which results in a larger number of hops for sending the message to the destination. In contrast, given a larger transmission range (e.g., 150 m), the number of hops of these three schemes are close, since the routing procedure can expedited by a larger communication coverage and more appropriate relay vehicles. Because of the random movement, Figure 11 (top) shows that there is no significant differences among the three schemes with regard to the connection stability (Figure 11 (top left)) and the frequency of link up/down (Figure 11 (top right)). However, with varying the vehicle speed, Figure 12 (top) shows that with the distance/angle information, the proposed HDTC scheme owns better group stability compared with other two schemes in terms of connection time (Figure 12 (top left)) and the frequency of the link up/down (Figure 12 (top right)). Observe that given a cone angle and a transmission range, the vehicle speed does not have a signified impact on the topology control (Figure 12 (bottom left)) and the routing task (Figure 12 (bottom right)) with respect to the three schemes.

Considering the impact of the cone angle and random movement, Figure 13 shows that a smaller cone angle leads to a smaller group size (Figure 13 (top left)), a smaller connection time (Figure 13 (top right)) and a larger number of hops for information routing (Figure 13 (bottom right)). In contrast, a larger cone angle improves connection stability and group formation. Thus, reasonable values of the cone angle may be applied in order to maintain the local topology structure. For Scenario 1, 60° ≤ *θ* ≤ 75° may be an appropriate range for the proposed scheme.

#### 6.2.2. Scenario 2

**Figure 14.**
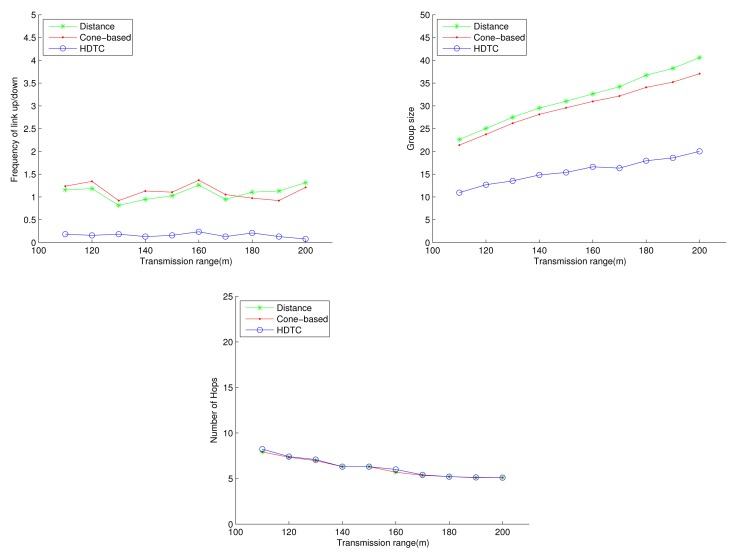
Scenario 2: The frequency of the link up/down, the group size and the number of hops with varying the connection distance.

**Figure 15.**
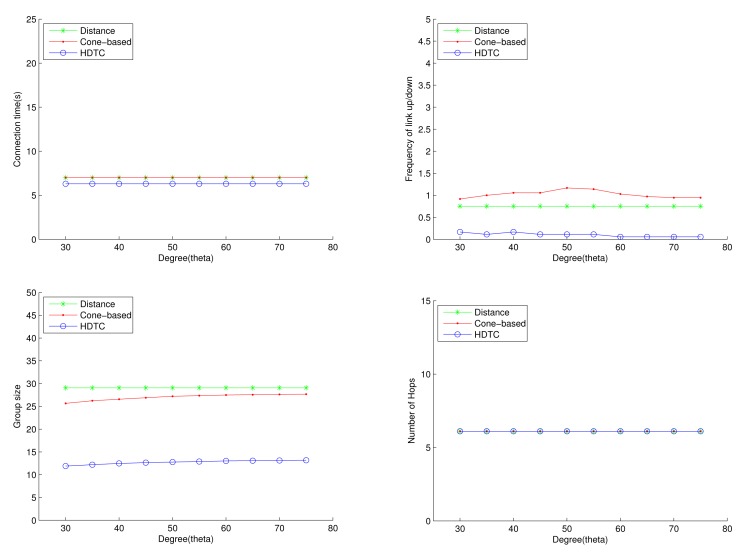
Scenario 2: The connection time, the frequency of the link up/down, the group size and the number of hops with varying *θ*.

As shown in Figures 14 and 15, compared with the distance scheme and the cone-based scheme, the proposed HDTC scheme is able to achieve a smaller group size, a more stable group formation and nearly the same connection time and number of hops for information dissemination. For the case of a straight-line motion (as shown in Figure 10 (top left)), with varying the transmission range, the HDTC scheme is superior to the other two schemes with regard to the group size (Figure 14 (top right)) and the number of in/out members (Figure 14 (top left)). Similarly, with varying the cone angle *θ*, the HDTC method in group members is about 55%∼65% less the number of members than the other two methods (Figure 15 (bottom left)); the HDTC method in the number of in/out members is about 80%∼90% less than the other two methods (Figure 15 (top right)).

**Figure 16.**
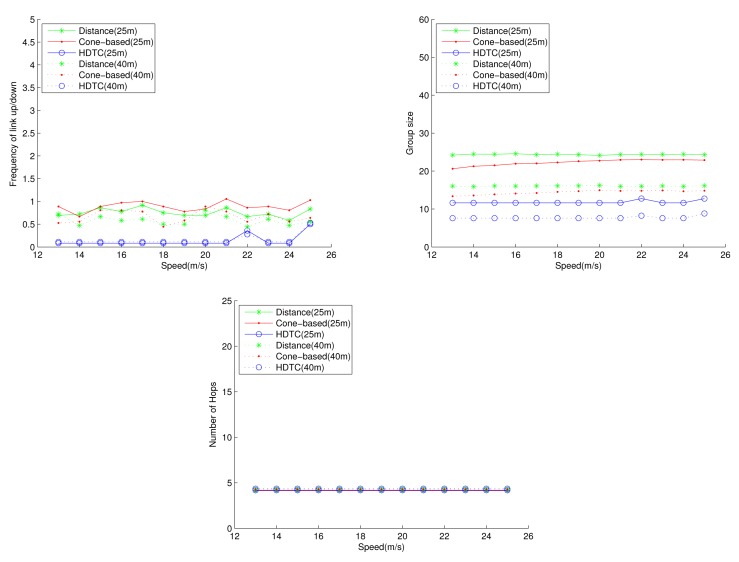
Scenario 2: The connection time, the frequency of the link up/down, the group size and the number of hops with varying the lane headway.

Notice that the above experiment is based on the land headway LH = 20 m. To evaluate the impact of the land headway (LH) on system performance, Figure 16 demonstrates the network behaviors with varying LH and the cone angle *θ*, which shows that a smaller value of LH may lead to a larger group size and a higher frequency of the link up/down. Compared with the cone-based method and the distance method, the HDTC method owns a lower frequency of the link up/down (Figure 16 (top right)) and a smaller group size (Figure 16 (bottom left)), due to its more stable network structure. Moreover, if LH < R, the number of hops for information routing is nearly independent of the values of LH. That is, for the HDTC scheme, the variation of the LH does not have a significant influences on system performance under this scenario.

#### 6.2.3. Scenario 3

**Figure 17.**
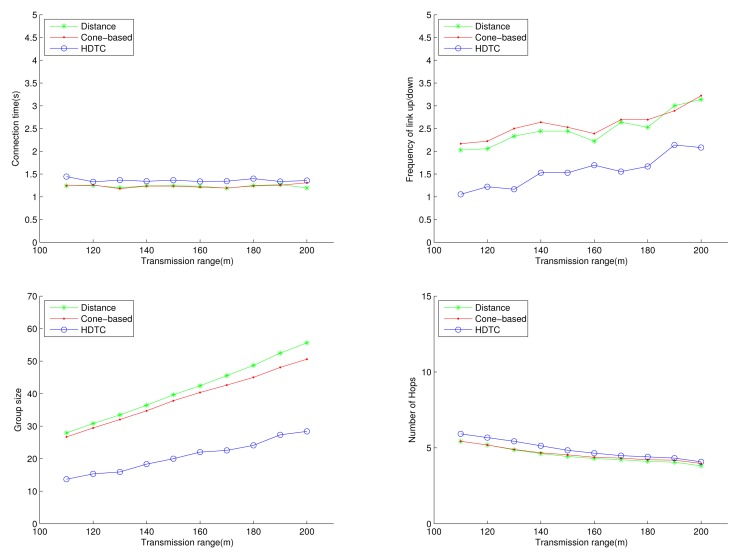
Scenario 3: The connection time, the frequency of the link up/down, the group size and the number of hops with varying the connection distance.

**Figure 18.**
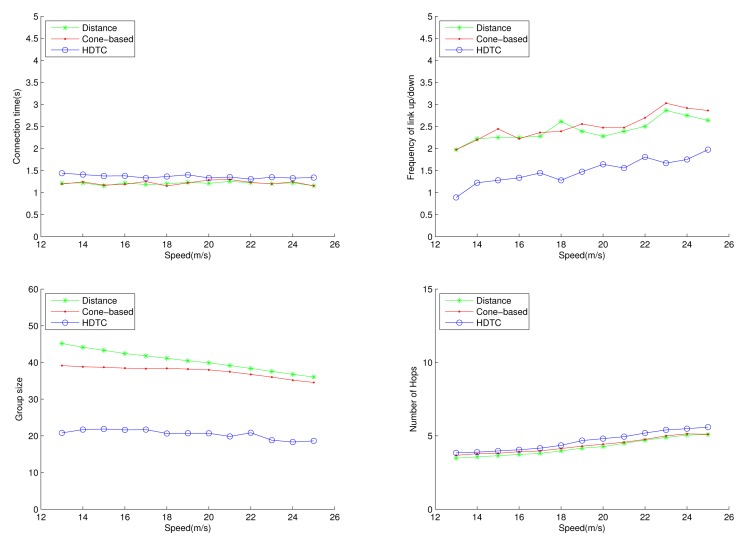
Scenario 3: The connection time, the frequency of the link up/down, the group size and the number of hops with varying the vehicle speed.

**Figure 19.**
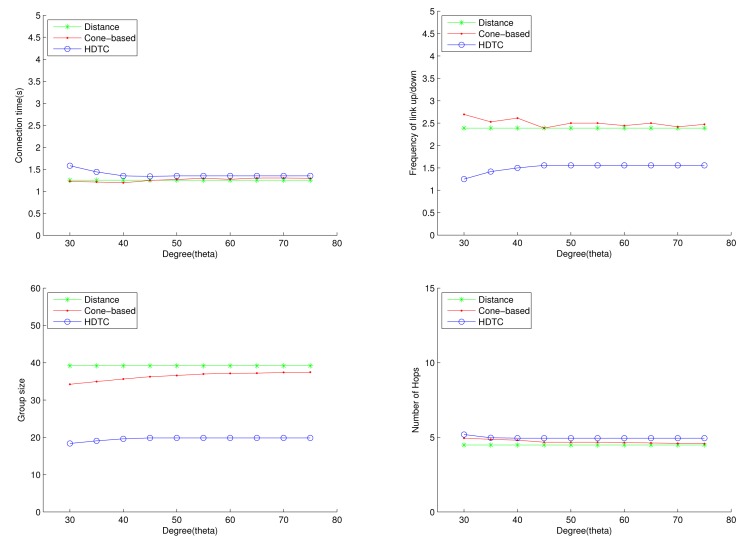
Scenario 3: The connection time, the frequency of the link up/down, the group size and the number of hops with varying *θ*.

For an intersection scenario, Figures 17, 18 and 19 show that in terms of the group size and the frequency of group reformation, the performance of the HDTC scheme is superior to the other two schemes. For instance, with varying the cone degree, the HDTC method in group members is about 60% less for the number of group members than the other two methods (Figure 19 (bottom left)); the HDTC method in the number of in/out group members is 60%∼70% less than the other two methods (Figure 19 (top right)). Moreover, in Scenario 3, the proposed HDTC scheme provides a longer connection time, which may stabilize the group formation (Figure 19 (top left)). However, due to a smaller group size, compared with the other two schemes, the HDTC scheme introduces additional 1∼2 hops for sending the message to the sink (Figure 19 (bottom right)). Similar descriptions can be applied to the results depicted in Figures 17 and 18.

Although the system performances highly depend on the type of mobility model occurring in the network, in general, the longer connection time the main node (say, the vehicle *u*) has, the better it maintains the communication channel; the lower the frequency of the link up/down that the source node has, the more stable can the group be made [35]. Note that the above results suggest that different arguments may have different impacts on topology management. Thus, if we can acquire the traffic information in advance, some arguments may be adaptively adjusted to obtain a better network performance.

### 6.3. Performance of the Simplified Model

**Figure 20.**
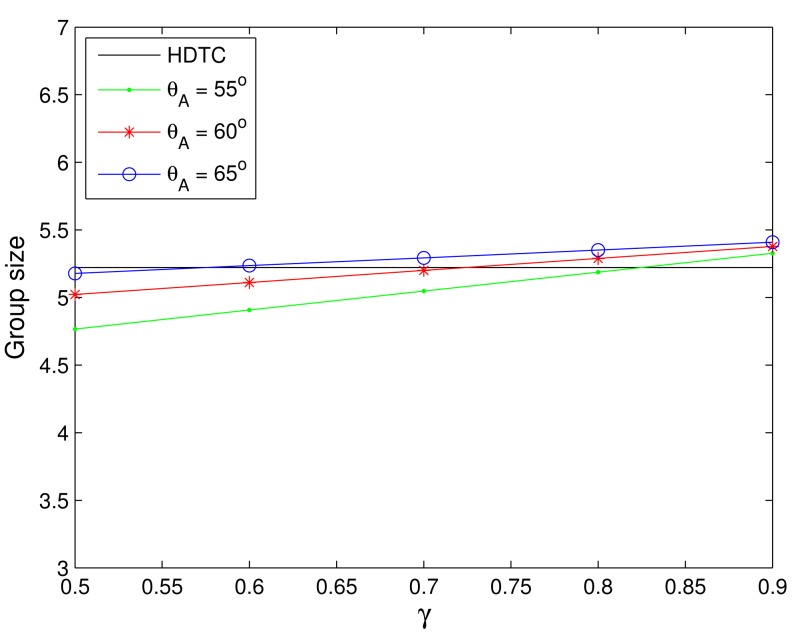
The average group size with varying *θ_a_* and the parameter *γ*.

The last set of experiments estimates the performance of the average number of communication group members with the PM model, as detailed in Section 4. With the simplified model and 500 typical runs, Figure 20 depicts the mean of the communication group size of the source vehicle considering random uniform deployment with varying the cone angle of Region A (*θ_A_*) and the parameter *γ*. Referring to Figure 20, we have the average group size |*G_u_*|^(PM)^ ≈ |*G_u_*|^(HDTC)^ with *θ_A_* = 65° and *γ* = 0.6, *θ_A_* = 60° and *γ* = 0.7, and *θ_A_* = 55° and *γ* = 0.8, respectively, which suggest that in order to improve the estimation accuracy, a larger value of parameter *γ* may be used in a smaller Region A (*i.e.*, a smaller *θ_A_*), such that the similarity measures in Region B are emphasized. The variation of the prediction achieves ‖*G_u_*|^(PM)^ − |*G_u_*|^(HDTC)^| ≤ 2.

Notice that with the probabilistic model (PM), the performance of the HDTC is roughly predicted, due to flexibility and similarity measures of the vehicles within the observation cone, suggesting that the PM model may provide a good approximation to the HDTC. Thus, the proposed simplified model may reasonably abstract the network behaviors and measure the system performance with the settings of the cone angle of Region A (*θ_a_*) and the parameter *γ*.

### 6.4. Analysis of Communication Complexity

This subsection evaluates the communication complexity of the proposed HDTC scheme, given a random environment (Scenario 1) with *θ* = 60^o^, transmission range = 150 m and an average speed = 21 m/s.

Referring to the simulation result (Figure 13 (bottom left)), the average communication overheads in the proposed HDTC method can be properly approximated, which yields the following:

- Source node sends a message to the communication group members (average group size = 14).
  $$N_{T}=1,N_{R}=14$$
- The communication group members reply a message to the source node.
  $$N_{T}=14,N_{R}=14$$
- Routing node selection:
  $$N_{T}=1,N_{R}=1$$
- The relay node replies a message to the source node, which completes the operations in one round.
  $$N_{T}=1,N_{R}=1$$
- Five rounds are performed in a typical run (*i.e*., the average number of hops = 5).

Thus, the total number of transmissions and receptions are 85 and 150, respectively.

**Figure 21.**
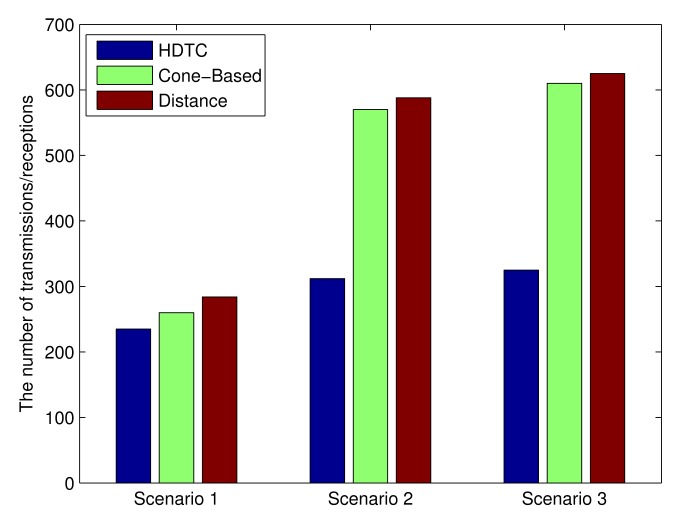
The communication complexity of the HDTC scheme, cone-based scheme and distance scheme under Scenarios 1, 2 and 3, respectively.

Similarly, given the same traffic scenario and referring to Figure 13 (bottom left)), the cone-based method [9] induces a group size = 20, and the number of hops = 4, which generates a total number of transmissions and receptions, 92 and 168, respectively. For the distance method [8], the typical run forms a group size = 22, and the number of hops = 4, which generates a total number of transmissions and receptions, 100 and 184, respectively. A similar analysis can be applied to Scenarios 2 and 3. Referring to Figure 21, the proposed HDTC method is shown to have lower communication complexity compared to the other two methods.

## 7. Conclusions

In this work, a hybrid distributed topology control scheme for vehicular sensor networks is presented. The proposed local management scheme investigates the key features of topology construction and maintenance, such as the group size, the degree of the cone, the connection time and the number of hops for message dissemination. With angle and distance information, the proposed HDTC scheme is able to achieve group stability and adaptability in vehicular sensor networking systems, as demonstrated in experimental results. Compared with the cone-based scheme and the distance scheme, the proposed HDTC provides a superior self-configuring technique, which can be applied to provide efficient distributed management in vehicular sensor networks. Future plans will involve generalizing the scheme to more realistic mobility models and investigating the impacts of the constraints of group size (e.g., the upper bound and the lower bound of the group size) on the system performance, so that the load and overhead of each communication group can be balanced.

## Figures and Tables

**Table 1.** Procedures of the HDTC scheme and information routing.

Phase I: Initial Selection	

(1)	Each vehicle broadcasts a Hello message for neighbor discovery.
(2)	Modified cone-based method:
	(a) Determine Group 1 members with angle information.
	(b) *G*_1_ = {*p′*|*p′* ϵ *N_u_*, cos *X*_(_*_p′_*_)_ ≤ cos *θ*}.
(3)	Proposed time-slot method:
	(a) Determine Group 2 members with distance information.
	(b) *G*_2_ = {*p′*|*p′* ϵ *N_u_, D_up'_*(*t*_0_) ≤ *d*_0_}.
(4)	Hybrid scheme
	(a) Determine the communication group for vehicle *u*.
	(b) *G_u_* = {*p′*|*p′* ϵ (*N_u_* ∩ *G*_1_ ∩ *G*_2_}.

Phase II: Routing Member Selection	

(1)	Determine the routing group members:
	(a) *R_u_* = {*p′*|*p′* ϵ *G_u_*, cos *Y*_(_*_p_*_′)_ ≤ cos *θ′*}
(2)	Determine the relay member:
	(a) *p′* = arg min*_p′_*_ϵ__*R*_1__ *D_p′_*_sink_

Phase III: Reforming *G_u_* and *R_u_*	

(1)	Communication group:
	(a) *R_u_* = {*p*′|*p*′ ϵ *G_u_*, cos *Y*_(_*_p′_*_)_ ≤ cos *θ′*}
(2)	Reselection of the relay member:
	**If** (*R_u_* == Ø),
	*p′* = arg min*_p′_*_ϵ_ *_G_u__* *D_p′_*_sink_,
	**else**
	*p′* = arg min*_p′_*_ϵ_ *_R_u__* *D_p′_*_sink_.
	**end**

**Table 2.** The values of simulation parameters.

	*θ*	**Transmission Range**	**Average Speed**
Default	75°	150 m	21 m/s
Angle variation	30°∼75°	150 m	21 m/s
Distance variation	75°	110∼200 m	21 m/s
Speed variation	75°	150 m	13∼26 m/s

## References

[1] Lin K. (2013). Research on adaptive target tracking in vehicle sensor networks. J. Netw. Comput. Appl..

[2] Santos R.A., Edwards A., Edwards R., Seed L. (2005). Performance evaluation of routing protocols in vehicular ad hoc networks. Int. J. Ad Hoc Ubiquitous Comput..

[3] Namboodiri V., Agarwal M., Gao L. A study on the feasibility of mobile gateways for vehicular *ad hoc* networks.

[4] Nadeem T., Shankar P., Iftode L. A Comparative Study of Data Dissemination Models for VANETs.

[5] Luo Y., Zhang W., Hu Y. A New Cluster Based Routing Protocol for VANET.

[6] Rathore N.C., Verma S., Verma S., Tomar G.S. CMAC: A Cluster Based MAC Protocol for VANETs.

[7] Hafeez K.A., Zhao L., Liao Z., Ma B.N.-W. Reliability of Cluster-Based Multichannel MAC Protocols in VANETs.

[8] Li L., Halpern J.Y., Bahl P., Wang Y.-M., Wattenhofer R. (2005). A Cone-Based Distributed Topology-Control Algorithm for Wireless Multi-Hop Networks. IEEE/ACM Trans. Netw..

[9] Zhao L., Lloyd E., Ravi S.S. Topology Control for Constant Rate Mobile Networks.

[10] Gharavi H. (2006). Control based mobile *ad hoc* networks for video communications. IEEE Trans. Consum. Electron..

[11] He Z.J. Structure Based or Structure Free? Topology Management in VANETs.

[12] Huang C.-C., Chiu Y.-H., Wen C.-Y. Using Hybrid AOA/TOA Information for Distributed Topology Control in VANETs.

[13] Zhang L.R., El-Sayed H. (2012). A Novel Cluster-Based Protocol for Topology Discovery in Vehicular *Ad Hoc* Network. Procedia Comput. Sci..

[14] Amis A.D., Prakash R., Vuong T.H.P., Huynh D.T. Max-min d-cluster formation in wireless *ad hoc* networks.

[15] Vodopivec S., Bester J., Kos A. (2014). A Multihoming Clustering Algorithm for Vehicular *Ad Hoc* Networks. Int. J. Distrib. Sens. Netw..

[16] Rawashdeh Z.Y., Mahmud S.M. (2012). A novel algorithm to form stable clusters in vehicular *ad hoc* networks on highways. EURASIP J. Wirel. Commun. Netw..

[17] Wen C.-Y., Tang H.-K. (2009). Autonomous Distributed Self-Organization for Mobile Wireless Sensor Networks. Sensors.

[18] Lehsaini M., Guyennet H., Feham M. A novel cluster-based self-organization algorithm for wireless sensor networks.

[19] Rappaport T.S. (2002). Wireless Communications: Principles and Practice.

[20] Yu J.Y., Chong P.H.J. (2005). A survey of clustering schemes for mobile ad hoc networks. IEEE Commun. Surv. Tutor..

[21] Dressler F. (2007). Self-Organization in Sensor and Actor Networks.

[22] Li M., Li Z., Vasilakos A.V. (2013). A Survey on Topology Control in Wireless Sensor Networks: Taxonomy, Comparative Study, and Open Issues. Proc. IEEE.

[23] Ramakrishnan, Rajesh R.S., Shaji R.S. (2011). CBVANET: A Cluster Based Vehicular *Ad Hoc* Network Model for Simple Highway Communication. J. Adv. Netw. Appl..

[24] Sharef B.T., Alsaqour R.A., Ismail M. (2014). Vehicular communication *ad hoc* routing protocols: A survey. J. Netw. Comput. Appl..

[25] Fu Q., Zhang L., Feng W., Zheng Y. Dawn: A density adaptive routing algorithm for vehicular delay tolerant sensor networks.

[26] Lee U., Magistretti E., Gerla M., Bellavista P., Corradi A. (2009). Dissemination and harvesting of urban data using vehicular sensing platforms. IEEE Trans. Veh. Technol..

[27] Choi O., Kim S., Jeong J., Lee H.-W., Chong S. Delay-Optimal Data Forwarding in Vehicular Sensor Networks.

[28] Gao H., Utecht S., Patrick G., Hsieh G., Xu F.Y., Wang H.D., Li Q. (2010). High speed data routing in vehicular sensor networks. J. Commun..

[29] Li F., Wang Y. (2007). Routing in vehicular ad hoc networks: A survey. IEEE Veh. Technol. Mag..

[30] Li F., Zhao L., Fan X.M., Wang Y. (2012). Hybrid Position-Based and DTN Forwarding for Vehicular Sensor Networks. Int. J. Distrib. Sens. Netw..

[31] Shao Y., Liu C., Wu J., Weigle M., Olariu S. (2009). Delay tolerant networks in VANETs. Handbook on Vehicular Networks.

[32] Billingsley P. (1979). Probability and Measure.

[33] Yu D., Li H., Gruber I. Path availability in ad hoc network.

[34] Teh S.K., Mejias L., Corke P., Hu W. Experiments in Integrating Autonomous Uninhabited Aerial Vehicles (UAVs) and Wireless Sensor Networks.

[35] Akhtar N., Ozkasap O., Ergen S.C. VANET Topology Characteristics under Realistic Mobility and Channel Models.

